# Genome-Wide Identification and Characterization of Vacuolar Processing Enzyme Gene Family and Diverse Expression Under Stress in Apple (*Malus* × *Domestic*)

**DOI:** 10.3389/fpls.2020.00626

**Published:** 2020-05-26

**Authors:** Jianfei Song, Fei Yang, Mi Xun, Longxiao Xu, Xiaozhi Tian, Weiwei Zhang, Hongqiang Yang

**Affiliations:** ^1^College of Horticulture Science and Engineering, Shandong Agricultural University, Tai’an, China; ^2^State Key Laboratory of Crop Biology, Shandong Agricultural University, Tai’an, China

**Keywords:** Genome-wide analysis, VPE gene family, diverse expression, salt stress, apple (*Malus* × *domestic*)

## Abstract

Vacuolar processing enzymes (VPEs) play an important role in stress resistance and development of plants. Despite their diverse roles, little information is available in apple (*Malus* × *domestic*). This study firstly presents the genome-wide identification of VPE family genes in apple, resulting in 20 family members those are unevenly distributed across six out of the 17 chromosomes. Phylogenetic analysis assigned these genes into four groups. Analysis of exon–intron junctions and motifs of each candidate gene revealed high levels of conservation within and between phylogenetic groups. *Cis*-element including w box, ABRE, LTR, and TC-rich repeats were found in promoters of *MdVPEs*. NCBI-GEO database shown that the expression of *MdVPEs* exhibited diverse patterns in different tissues as well as the infection of *Pythium ultimum* and *Apple Stem Grooving Virus*. Furthermore, qRT-PCR showed that *MdVPE* genes were responsive to salt, cadmium, low-temperature, and drought. Overexpression of MDP0000172014, which was strongly induced by salt and drought stress, significantly decreased *Arabidopsis* tolerance to salt stress. The genome-wide identification and characterization of *MdVPEs* in apple provided basic information for the potential utilization of *MdVPEs* in stress resistance.

## Introduction

Programmed cell death (PCD), which plays an important role in developmental processes and stress resistance of eukaryotes, is a genetically regulated physiological process (Hatsugai et al., 2015). In animals, the performed of PCD requires the caspase enzymes with cysteine-dependent aspartyl protease activity (Fagundes et al., 2015), and caspase enzymes could hydrolyze the substrates with specific aspartic acid residues (Lamkanfi et al., 2002; De Pinto et al., 2012). Unfortunately, its protein sequences are not conserved in the genomes of plants (Lam and Zhang, 2012). However, caspase-like enzymatic activity has been shown that is essential in many forms PCD of plants (Del Pozo and Lam, 1998; Woltering et al., 2002; Baskett, 2012). And some caspase-like proteases including subtilases (Vartapetian et al., 2011), metacaspases (Tsiatsiani et al., 2011) and legumains (Rojo et al., 2004) had been identified one after another.

Legumain, which is referred to as vacuolar processing enzymes (VPEs), has the characteristics of caspase-1-like activity (Hatsugai et al., 2015). VPEs is conversed in plants, and their conversed functional domain is peptidase_C13 (Pfam ID: PF01650), which composed of CASc superfamily motifs (Vorster et al., 2019). Based on the expression and functional characteristics, VPEs could be divided into three sub-families: vegetative VPEs, seed VPEs, and uncharacterized VPEs. Vegetative VPEs including γVPE and δVPE, are essential for stress resistance of plants, while seed VPEs including αVPE and βVPE, play an important role in developmental process (Christoff et al., 2014), and the functions of uncharacterized VPEs have not been identified.

As a key regulator of plant cell death, VPEs were involved in many plant developmental processes. For example, *NtTPE8*, a VPE-like protease from tobacco, only expressed in the integumentary tapetum of tobacco seeds, and its downregulation induced seeds abortion (Wang et al., 2019). Heterologous expression of *VvβVPE*, a β*VPE* from *Vitis vinifera*, was essential for ovule maturation and the increased germination of seeds in *Arabidopsis* (Gong et al., 2018). Expression of *IbVPE1* from sweet potato in *Arabidopsis* affected leaf development, flowering time and chlorophyll catabolism (Jiang et al., 2019). Previous study also reported VPEs were involved in the regulation of seed size in barley (Radchuk et al., 2018; Yamada et al., 2019), the breakdown of apical bud dominance in potato tubers (Teper-Bamnolker et al., 2012), the development and senescence of root nodules (Van Wyk et al., 2014; Cilliers et al., 2017), and the floral bud abortion of radish (Zhang et al., 2013).

VPEs were also involved in the resistance of plants biotic and abiotic stress. VPEs has been identified to be essential for TMV-induced hypersensitive cell death of *Nicotiana benthamiana* by VIGS (virus-induced gene silencing) (Hatsugai et al., 2004) and mycotoxin-induced cell death in *Arabidopsis* (Kuroyanagi et al., 2005). *γVPE* promoted the heat shock-induced cell death in *Arabidopsis* leaves (Li et al., 2012). VPEs were also involved in aluminum-induced cell death (Kariya et al., 2013, 2018) and H_2_O_2_-induced cell death (Deng et al., 2011; Kim et al., 2014). Besides, our lab also found that *MhVPE*γ, a *γVPE* isolated from apple rootstock-*Malus hupenensis* Rhed., was involved in cadmium-induced cell death (Ran et al., 2014; Zhang et al., 2019) and high temperature-induced cell death (Su et al., 2015).

Consider that so many important roles of VPEs, they have been analyzed at the whole genome scale in various model plants and crops (Vorster et al., 2019). However, the study related to them in apple, one of the fruits with the largest production and consumption in the world, is little. Fresh roots of apple have abundant vacuole, an organelle plays an important role in the stress resistance of plants, and VPE is essential for function performed of the vacuole (Shimada et al., 2018). Furthermore, genome-wide identification and characterization of a gene family can provide basic information for the potential utilization of the gene function. Since apple genome sequencing (Velasco et al., 2010), more and more gene family of apple had been identified (Meng et al., 2016; Mao et al., 2017), whereas the whole information of VPE family genes in apple is limited.

In this study, 20 VPE genes were identified in the apple (*Malus* × *domestic*) genome, and their expression profiles were determined in different tissues, and during the infection of *Pythium ultimum* and *Apple Stem Grooving Virus* (ASGV). qRT-PCR was also used to detect the expression levels in response to salt, cadmium, drought and low-temperature treatment. Overexpression of MDP0000172014, which was strongly induced by salt and drought stress, significantly decreased *Arabidopsis* tolerance to salt stress and promoted salt stress-induced roots cell death. Our widely identified and expression analysis of *MdVPEs* in apple filled the blank of woody plants VPE information and contributed to the further exploration of VPE functions.

## Materials and Methods

### Plant Materials and Stress Treatment

“Royal gala” tissue culture seedlings were used in this study. Consistent growth tissue culture seedlings were selected and rooted with Murashige and Skoog (MS) agar medium which contain 30 g/L sucrose and 0.3 mg/L IBA. After grown for 30 days, the rooted tissue culture seedlings were transferred to ^1^/_2_ Hoagland’s nutrient solution for stress treatment. For salt stress, ^1^/_2_ Hoagland’s nutrient solution with 200 mM NaCl was used for plant treatment; for cadmium stress, ^1^/_2_ Hoagland’s nutrient solution with 200 μM CdSO_4_ was used for plants treatment; for low-temperature stress, illumination incubator with 4°C and ^1^/_2_ Hoagland nutrient solution was used for plants treatment, and for drought stress, ^1^/_2_ Hoagland’s with 5% PEG 6000 (W/V) was used for plants treatment. The plants treated with ^1^/_2_ Hoagland’s nutrient at room temperature (RT) was as the control of each group treatment. Each group treatment was treated for 24 h. After 24 h of such pre-cultivation, stress treatments were initiated. All collected tissue samples had treated for 24 h were frozen quickly in liquid nitrogen and stored at −80°C for RNA extraction.

### Identification of Vacuolar Processing Enzyme Family in Apple

The Hidden Markov Model (HMM, PF01650) of VPEs was downloaded from the Pfam database^1^ and used to search for the apple genome database by HMM 3.0. To avoid the loss of members due to the incomplete domain of VPE, local BLAST-P searches were performed in Phytozome^2^ plant genome database using the protein sequences of *Arabidopsis* (AT1G62710.1, BETA-VPE; AT2G25940.1, ALPHA-VPE; AT3G20210.1, DELTA-VPE, and AT4G32940.1, GAMMA-VPE), which downloaded from TAIR.^3^ And then combined the two-part results and deleted the repeated sequences. The results were submitted to SMART,^4^ NCBI–CDD^5^ (Marchler-Bauer et al., 2017) and Pfam to identify the protein structure domain. The sequences which do not contain the peptidase_C13 domain and no complete Open Reading Frame (ORF) were deleted.

### Sequence Annotation and Structural Analysis

All the high confidence sequences were submitted to the ExPaSy Proteomics Server online tools^6^ for predicting molecular weight (MW), isoelectric point (PI) and amino acid (AA) number. The chromosomal location and exons quantity information were downloaded from the Phytozome. The exon-intron structures of *MdVPE* genes were visualized via Gene Structure Display Server 2.0^7^ (GSDS) (Hu et al., 2015) and the conserved motifs of *MdVPEs* were analyzed by the MEME program version 5.0.2,^8^ with the parameter as 6 × 50 and the number of motifs as 10. ProtComp version 9.0^9^ was used to predict subcellular localization of *MdVPEs*.

### Phylogenetic Analysis, Chromosome Localization and Gene Duplication of *MdVPEs*

Multiple sequence alignment of AA sequences in *Arabidopsis* and apple were used the clustal W version 2.0 (Thompson et al., 1997) and the MEGA 7.0 (Kumar et al., 2016) was used to generate phylogenetic trees by neighbor-joining (NJ) method, with Bootstrap parameters were tested for 1,000 times. Chromosome location was shown by Map Draw version 2.1. Gene duplication of *MdVPEs* including tandem duplicated genes and segmental duplicated genes were identified by TBtools (Chen et al., 2018) with the *E*-value is 1e−10.

### Analysis of *Cis*-Acting Element in *MdVPEs’* Promoters

The upstream sequences (2 kb) of the *MdVPE*-coding sequences were retrieved from the Phytozome plant genome database and then submitted to Plant CARE^10^ to identify four regulatory elements, abscisic acid (ABA)-responsive elements (ABRE); low-temperature responsive elements (LRE); TC-rich repeats, which were involved in defense and stress response; and w box, which is the binding site of WRKY transcription factor-play an important role in defensing to drought stress.

### Expression Profile Analysis of *MdVPEs* in Different Tissue and During *P. ultimum* and ASGV via NCBI-GEO Database

Expression profile database (GSE42873, for different tissues; GSE62103, for *P. ultimum*; GSE53825, for ASGV) were downloaded from NCBI-GEO database.^11^ *MdVPE* sequences were used BLAST for probe database (GPL16374, for different tissues; GPL18137, for *P. ultimum* and ASGV) and the matched probes were selected to represent *MdVPEs*. Heatmaps showed the expression profiles of *MdVPEs* were constructed by TBtools (Chen et al., 2018) and RPKM (reads per kilobase of exon model per million mapped reads) values of each gene obtained in the NCBI-GEO were used to compare their expression levels between different tissues and during the infection of *P. ultimum* and ASGV.

### Total RNA Extraction and qRT-PCR

Total RNA was extracted from “royal gala” seedlings roots via polysaccharide rich polyphenols total RNA extraction kits (TianGen, China). First-strand cDNA was obtained by Prime Script^TM^ RT reagent Kit with gDNA Eraser (Perfect Real Time) (TaKaRa, Japan). qRT-PCR was performed three independent biological replicates on LightCycler^®^ 96 (Roche) using the TB Premix Ex Taq II (Tli RNaseH Plus) (TaKaRa, Japan) according to the specification. All primer sequences for qRT-PCR were given in Supplementary Table 1, and *Md-actin* was used as the internal control gene. The data were analyzed by 2^–ΔΔ*Ct*^ method (Livak and Schmittgen, 2001).

### *Arabidopsis* Transformation and Stress Treatments

Col-0 *Arabidopsis* were used for genetic transformation. Full-length MDP0000172014 was cloned from cDNA by using primers MDP0000172014-F/R (Supplementary Table 1), and its coding region was inserted into the pBI121 vector under the control of the 35S promoter through the In-Fusion Cloning (C112, Vazyme, China). The recombinant vector named pBI121-35S-MDP0000172014 was transformed into GV3101, an *Agrobacterium tumefaciens* strain. Inflorescence infection was used for the transformation of Col-0 *Arabidopsis* (Clough and Bent, 1998). The transgenic *Arabidopsis* seeds were sterilized and selected for 50 mg/L kanamycin resistance on MS agar medium and finally three independent third homozygous (T3) were generated.

The T3 and Col-0 *Arabidopsis* seeds were sterilized and growth on MS agar medium for 7 days, and then transferred onto MS agar medium containing 100 mM NaCl and cultured in an incubator for another 10 days. The seedlings were then photographed. The root length was recorded based on at least 10 seedlings. Seven-days-old-seedlings growth on standard MS agar medium were transferred onto a matrix containing 30% turf, 30% vermiculite, and 10% perlite and cultured in illumination incubator with culture conditions of 24°C, light cycle for 16 h light/dark 8 h, watered once every 4 days. Forty-days-old seedlings growth on a matrix which were watered with ^1^/_2_ Hoagland’s nutrient solution (as in the control) or ^1^/_2_ Hoagland’s solution containing 200 mM NaCl. The roots treated for 48 h and then sampled to measured MDA, H_2_O_2_ content and other physiological indexes.

### Measurement of H_2_O_2_ and MDA

H_2_O_2_ content was determined by sulfuric acid precipitation (Patterson et al., 1984). The MDA content was performed according to the thiobarbituric method and the absorbance of the mixture was measured at 532, 600, and 450 nm (Landi, 2017).

### Measurement of Cell Death Number and Vacuolar Processing Enzyme Activity

The measurement of cell death followed the Evans blue staining (Zhang et al., 2019). A total of 0.1 g roots were washed and placed in 0.25% (W/V) Evans blue solution. After cultured for 24 h, the roots were then rinsed with deionized water until no more blue stain eluted and put into a centrifuge tube containing 5 mL 1% SDS solution for 24 h to extract. A total of 1% SDS solution with unstained roots was used as negative control and the roots completely killed in boiling water 1 h were used as the positive control. The extracting solution was measured according to the absorbance of at 600 nm.

Vacuolar processing enzyme activity was measured following Wang et al. (2009) with slight modifications. VPE-specific substrate Ac-ESEN-MCA (Ac–Glu–Ser–Glu–Asn–MCA (Peptide Institute, Japan)) was used to measure the VPE activity. 0.1 g roots were used to make crude protein extract, and the extract was incubated with 100 μM Ac-ESEN-MCA in an acidic buffer (100 mM dithiothreitol, 100 mM sodium acetate, and pH 5.5) for 2 h at 20°C. The fluorescence was monitored under an excitation wavelength of 380 nm and an emission wavelength of 460 nm (Shimada et al., 2003; Wang et al., 2009).

### Statistical Analysis

SPSS software version 19.0 (IBM, Chicago, IL, United States) was used to analyze variance (ANOVA). All experiments were carried out in triplicate (*n* = 3) and expressed as mean value ± standard deviation. Duncan’s new complex range method was used for significance analysis with the test level was 5% (*P* < 0.05), and origin 2017 was used to draw the chart.

## Results

### Identification and Characterization of Vacuolar Processing Enzyme Gene Family in Apple

To identify VPE genes in the apple genome, the HMM of VPEs was downloaded from the Pfam database and used to search proteins database of apple by hmm search 3.0. Moreover, *Arabidopsis* VPE protein sequences were downloaded from the TAIR database and used to BLAST-P in the apple proteins database. Based on the SMART, NCBI-CDD, and Pfam, all of the *MdVPE* proteins without peptidase_C13 domain or complete ORF were removed. Finally, a total of 20 *MdVPE* genes in apple were obtained and annotated (Table 1). As shown in the Table 1, the size of these proteins ranges from 118 (MDP0000258293) to 780 (MDP0000241162) AAs residues, with the MW varies from 18.9 kDa (MDP0000292165) to 85.5 kDa (MDP0000241162) and the PI distribution varies from 5.51 (MDP0000172014 and MDP0000248773) to 9.65 (MDP0000256408). Subcellular locations were also predicted of *MdVPE* proteins by ProtComp version 9.0. Most of the *MdVPEs* were predicted to be vacuole proteins, whereas MDP0000243227 and MDP0000196515 were predicted to be endoplasmic reticulum (ER) membrane proteins. Unfortunately, MDP0000256408 and MDP0000292165 were predicted to be free of sub-cellular localization proteins.

**TABLE 1:** The information of *MdVPE* genes.

**Gene ID**	**Chr.**	**Genomic location**	**ORF**	**Exon**	**AA**	**MW (kDa)**	**pI**	**Subcellular localization**
MDP0000122571	4	5890702..5895897	1635	10	545	60.7	6.53	Vacuole
MDP0000256408	4	16299137..16309311	2166	10	721	80.3	9.65	NONE
MDP0000084203	8	11271539..11274898	1476	9	492	53.9	5.81	Vacuole
MDP0000241162	8	11247737..11259787	2340	13	780	85.5	6.98	Vacuole
MDP0000937205	9	13845992..13848285	1401	9	467	52.3	8.59	Vacuole
MDP0000188488	14	15905312..15909775	1422	9	473	52.5	5.63	Vacuole
MDP0000165304	15	12750152..12752592	2441	8	394	43.9	9.36	Vacuole
MDP0000166283	15	12770357..12772708	1389	9	463	51.2	6.57	Vacuole
MDP0000172014	15	965933..969041	1485	9	495	54.3	5.51	Vacuole
MDP0000227138	15	12767658..12770005	1143	7	380	42.1	9.18	Vacuole
MDP0000227977	15	12755044..12755900	567	3	188	21.3	9.26	Vacuole
MDP0000248773	15	950583..953693	1485	9	494	54.3	5.51	Vacuole
MDP0000256148	15	12674295..12682480	1788	13	596	66.7	7.95	Vacuole
MDP0000288837	15	12748457..12752533	1185	8	394	44.0	9.30	Vacuole
MDP0000292815	15	9169989..9171921	1494	5	498	54.3	6.25	Vacuole
MDP0000321943	15	12754968..12757312	1383	9	461	51.0	6.43	Vacuole
MDP0000759605	15	12803042..12804258	795	5	265	28.8	5.57	Vacuole
MDP0000292165	17	23097979..23101738	519	4	172	18.9	8.59	NONE
MDP0000243227	Unanchored	24749900..24753170	1206	10	401	45.4	5.57	Endoplasmic reticulum
MDP0000196515	4	21576985..21580173	1239	11	412	45.2	5.57	Endoplasmic reticulum

### Sequence Alignment and Phylogenetic Analysis of *MdVPE* Genes

**FIGURE 1:**
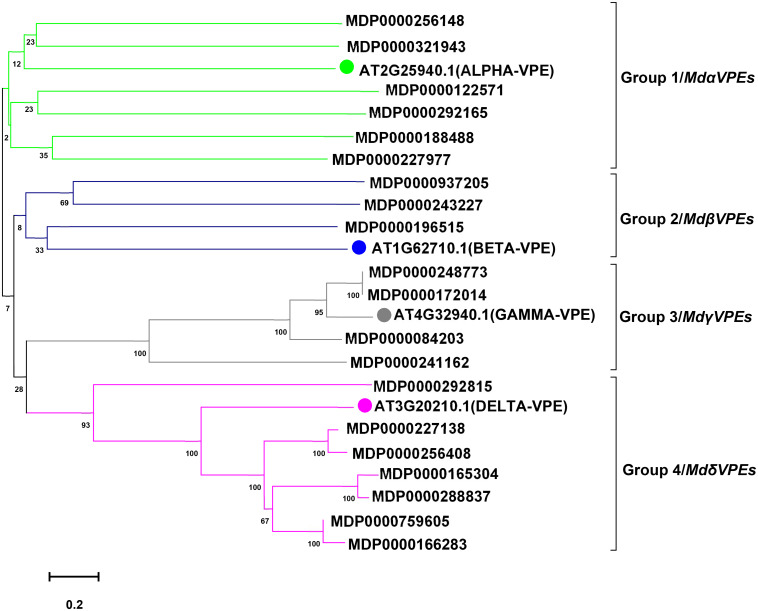
Phylogenetic analysis of apple and *Arabidopsis* VPE proteins. A total of 20 VPE proteins from apple and four from *Arabidopsis* were aligned using Clustal W. The phylogenetic tree was constructed using the MEGA 7.0 program by the neighbor-joining (NJ) method with bootstrap values 1,000 using protein sequences. According to *Arabidopsis* VPE protein sequcences, *MdVPE* proteins were devided into four groups. Different colors represent different groups.

To predict the potential function of *MdVPEs*, we constructed an unrooted phylogenetic tree by MEGA 7.0 following the NJ method. with the imported *Arabidopsis* VPE proteins as the reference proteins, *MdVPE* proteins were divided into four groups, and each group contains diverse *MdVPEs* family members (Figure 1). Group 4 had the most members (seven), followed by group 1 (six), while group 3 had four. Group 2 has the smallest, only three members.

### Structural Characterization Analysis of *MdVPEs*

**FIGURE 2:**
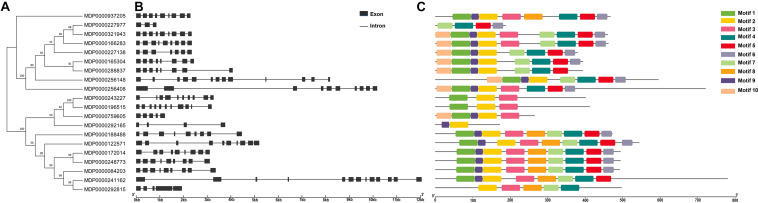
Phylogenetic relationship, gene structure, conserved motif and proteins structure analysis of *MdVPE* genes. **(A)** Phylogenetic tree of 20 *MdVPE* proteins. The unrooted neighbor-joining phylogenetic tree was constructed with MEGA 7.0 using full-length amino acid sequences of 20 *MdVPE* proteins, and the bootstrap test replicate was set as 1,000 times. **(B)** The exon-intron structure of *MdVPE* genes. Black boxes represent exons and black lines represent introns. **(C)** Distributions of conserved motifs in *MdVPE* genes. Ten putative motifs are indicated in different colored boxes. For details of motifs refer to Table 2.

To identify the structure difference between *MdVPEs*, the gene and protein structure of *MdVPEs* were visualization by GSDS 2.0 and the MEME program. Most of the *MdVPEs* have the exons above five (including five) (90%), with only two members have less than five exons (MDP0000227977, three; MDP0000292165, four), and all the members contain introns (Figure 2B). The similar exon-intron structure of *MdVPEs* suggested that they has a closely evolutionary relationship and similar functions.

**TABLE 2:** List of putative motifs of *MdVPE* proteins.

**Motif**	**Width**	**Best possible match**
1	50	KRWAVLVAGSNGYYNYRHQADICHAYQJLKKGGLKDENIIV FMYDDIAYN
2	50	VPKDYTGDHVNARNLYAVILGBKSALTGGSGKVLSSGPNDH VFIYYADHG
3	50	YVYANDLIEVLKKKHASKGYKSMVFYJEACESGSIFEGLLP ENLNIFATT
4	50	ISRAVBQRDTKLLYFQQKLQRAPTGSQEKQGAQKQLLLEIA HRKHVDYSI
5	41	LFGHEKSSNVLMNVRPQGQPVVDBWDCFKNFLNI YEKYCGH
6	29	GMKYTRAIANICNAGITTEKMVA ASDQTC
7	41	VRNRGTGSHVMQYGDMSHKKEFLFAYMGTBPSNR SHTSTGD
8	50	WGTYCPGEYPSPPPEYDTCLGDLYSVAWMEDSDIHNLKSE TLHQQYELVK
9	21	SENPRKGVIINKPNGHDVYKG
10	41	HGYCGVLLSLTLLSLAIHGSFCFPEINGDNKGSP RTTTDKG

Ten conserved motifs of *MdVPEs* were identified by the MEME program, whose size ranged between 29 to 50 AA residues. Based on the NCBI-CDD, except the motif four, five and six is unknown, the others belong to the CASc superfamily, which is an important part of the Peptidase C_13 domain. Among the *MdVPEs* proteins, motifs five and seven near the *N* terminal, while motifs three and six near the *C* terminal and the motifs of all *MdVPEs* proteins are highly conserved (Figure 2C). In addition, all motifs details were listed in Table 2.

### Chromosomal Locations and Genes Duplicated of the *MdVPEs*

**FIGURE 3:**
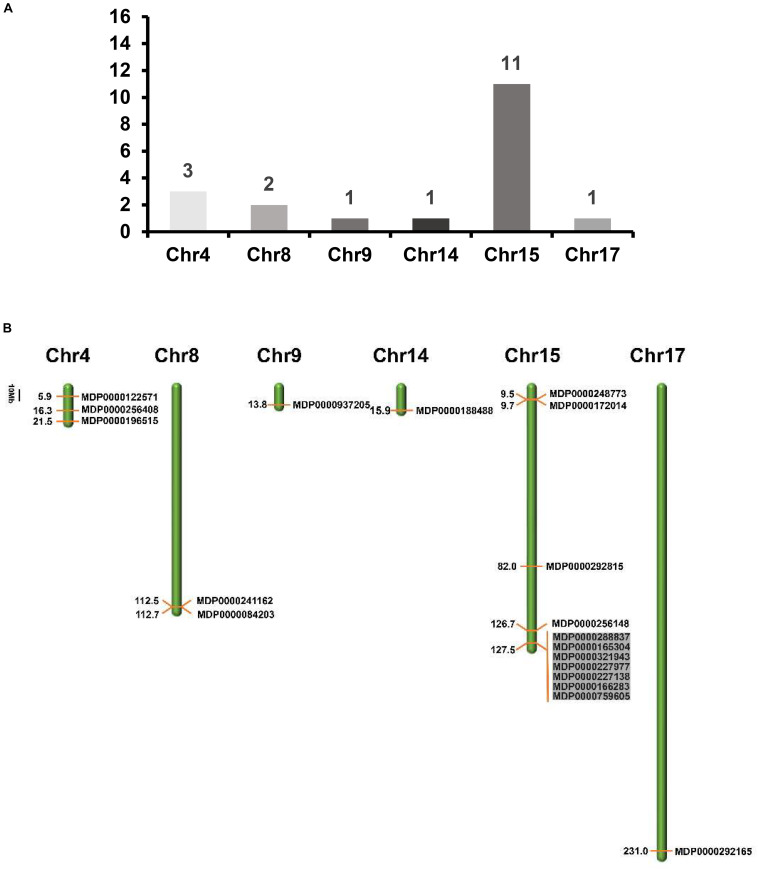
The distribution of *MdVPE* genes in apple chromosomes. **(A)** The number of *MdVPE* genes in each chromosome. **(B)** *MdVPEs* were mapped onto apple chromosomes. The black boxes represented clusters of genes.

To determine the distribution of *MdVPE* genes in 17 apple chromosomes, we analyzed their chromosomal location by MapDraw version 2.1. The *MdVPE* genes were spread among six chromosomes of the apple, including chromosome 4, 8, 9, 14, 15, and 17. Among them, chromosome 15 contains the most of *MdVPE* genes, followed by chromosome 4 (three) and chromosome 8 (two), whereas chromosome 9, 14, and 17 had only one *MdVPEs* gene (Figure 3A). The genetic distance of*MdVPEs* genes was shown in Figure 3B and the *MdVPE* genes cluster were formed in chromosome 15 (black boxes). The uneven distribution of *MdVPE* genes on chromosomes indicated that genetic variations exist in the evolutionary process of apple.

During the evolutionary process of apple, genes duplication contributed to the generation of a gene family. Among them, segmental duplication played the most important role, followed by tandem duplication (Cannon et al., 2004). Thus, we analyzed the genes duplicated of the *MdVPEs*. Unfortunately, we have not found segmental duplication or segmental duplication in the *MdVPE* genes family. This suggested the expansion of the *MdVPE* genes family is slow.

### Stress-Related *Cis*-Elements in *MdVPEs* Promoters

**FIGURE 4:**
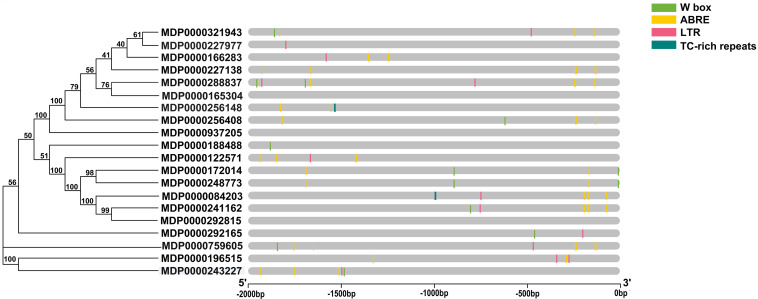
Predicted stress-related *cis*-elements in *MdVPE* promoters. Promoter sequences (–2,000 bp) of *MdVPEs* are analyzed by Plant CARE. Different color boxes represent different *cis*-element. According to the bottom scale, the upstream length to the translation start site can be inferred.

To predict the potential functions of *MdVPEs* under stresses, *cis*-element including w box, ABRE, LTR, and TC-rich repeats were analyzed by PlantCARE. All *MdVPEs* possessed at least one stress-response-related *cis*-element except MDP0000165304, MDP0000937205, and MDP0000292815, suggesting that the expressions of *MdVPEs* were associated with these abiotic stresses. In total, 14 *MdVPEs* (70.0%) had two or more ABREs, which suggested potential ABA response of *MdVPEs*, 10 *MdVPEs* (50.0%) had one or more w box, and this indicated *MdVPEs* may be involved in salt and drought stress. One or two LTRs existed in 11 *MdVPEs*, and one TC-rich repeats were found in two *MdVPEs* (Figure 4). The *cis*-element analysis indicated that *MdVPEs* may be involved in different abiotic stresses.

### Expression Profile of *MdVPEs* in Different Tissues

**FIGURE 5:**
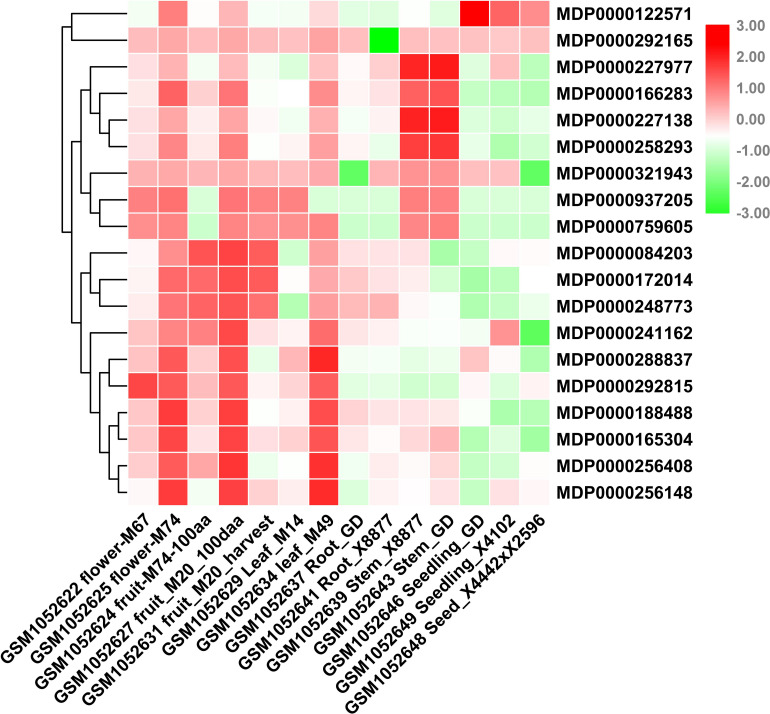
Expression profiles of *MdVPE* genes in different tissues of apple. NCBI-GEO data (GSE42873) were downloaded for the expression profile analysis. GSM1052622 flower-M67 and GSM1052625 flower-M74 represent the whole flower of M67 and the whole flower of M74, respectively. GSM1052624 fruit-M74-100aa, GSM1052627 fruit-M20-100aa, and GSM1052631 fruit-M20-harvest represent the 100 days after anthesis-fruit of M74, the 100 days after anthesis-fruit of M20 and fruit flesh at harvest of M20, respectively. GSM1052629 leaf-M14 and GSM1052634 leaf-M49 represents the whole leaf of M14, the whole leaf of M49, respectively. GSM1052637 Root-GD and GSM1052641 Root-X8877 represents the *in vitro* root of Golden Delicious, the *in vitro* root of X8877, respectively. GSM1052639 stem-X8877 and GSM1052643 stem-GD represent the fully developed-stem of X8877, the fully developed-stem of Golden Delicious, respectively. GSM1052646 seeding-GD and GSM1052649 seeding-X41002 represents the 10 days old-seedling of Golden Delicious, the 10 days old-seedling of X41002, respectively. GSM1052648 seed-X4442 × X2596 represents the dormant seed from cross X4442 and X2596. M67, M74, M20, M14, M49, X8877, Golden Delicious, X41002, X4442, and X2596 represent apple cultivars. RPKM values of *MdVPE* genes were transformed by log2 and the heatmap was constructed by TBtools.

Consider that any given gene functions is closely related to its expression patterns. Therefore, to provide more information for the study of *MdVPEs* potential function, we analyzed the expression patterns of 20 *MdVPEs* in different tissues including flowers, fruits, leaves, roots, stems, seeds, and seedlings based on NCBI-GEO (GSE42873) database. In the heatmap (Figure 5), red was used to represent a relatively high expression level, while the green was used to represent a relatively weak gene expression level. Some *MdVPEs* had similar expression patterns in various tissues. MDP0000241162, MDP0000288837, MDP0000292815, MDP0000188488, MDP0000165304, MDP0000256408, and MDP0000256418 has relatively higher expression levels in flowers, fruits, and leaves, but relatively lower in roots, stems, seeds, and seedlings. Although MDP0000122571, MDP0000227977, MDP0000166283, MDP0000227138, and MDP0000258293 also were relatively higher expression levels in flowers, fruits, and leaves, they had more higher expression levels in stems (MDP0000227977, MDP0000166283, MDP0000227138, and MDP0000258293) or seeds and seedlings (MDP0000122571). Additionally, MDP0000122571 and MDP0000321943 had not much different expression levels in various tissues. Furthermore, some *MdVPE* genes had higher expression levels in specific tissues. For example, MDP0000227977, MDP0000166283, MDP0000227138, and MDP0000258293 have the highest expression levels in stems, this suggested MDP0000227977, MDP0000166283, MDP0000227138, and MDP0000258293 may contributed to the function exertion of stem. Additionally, MDP0000122571 has the highest expression levels in seeds and seedlings, this implied MDP0000122571 may play a central role in the development of seeds and seedlings in apple. Interestingly, MDP0000084203, MDP0000172014, and MDP0000248773 (all belong to *MdγVPEs*) have the highest expression levels in roots, and this indicated they may be involved in the response to soil stresses or the development of roots. In summary, the different expression patterns of *MdVPEs* in various tissues suggested different *MdVPEs* played a different role in different tissues of apple.

### Expression Profile of *MdVPEs* With the Infection of *P. ultimum* and Apple Stem Grooving Virus

**FIGURE 6:**
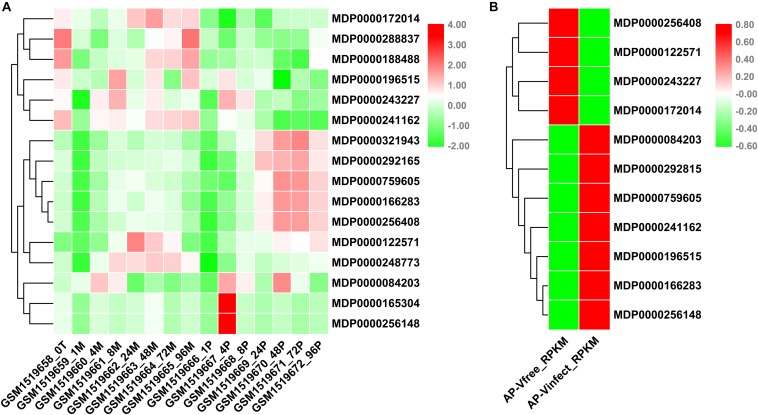
Expression profile of *MdVPEs* under *P. ultimum* and *Apple Stem Grooving Virus* (ASGV) stress. **(A)** Expression profile of *MdVPEs* under *Pythium ultimum* stress. NCBI-GEO data (GSE62103) were downloaded for the expression profile analysis. GSM1519658 represents the apple root organization at 0 h, GSM1519662, GSM1519663, GSM1519664, and GSM1519665 represents the simulated inoculation mold 24, 48, 72, and 96 h, and the apple root organization, GSM1519669, GSM1519670, GSM1519671, and GSM1519672 represents apple root tissue when inoculated with humus 24, 48, 72, and 96 h, respectively. **(B)** Expression profile of *MdVPEs* under ASGV stress. NCBI-GEO data (GSE53825) were downloaded for the expression profile analysis. AP-Vinfect represents ASGV-infected asymptomatic apple plantlets and AP-Vfree represents virus-free apple plantlets. RPKM values of *MdVPE* genes were transformed by log2 and the heatmap was constructed by TBtools.

VPEs had been reported play an important role in disease resistance of plants (Vorster et al., 2019). *P. ultimum* seriously threatened to the quality and yield of apples. To understand the potential involvement of *MdVPE* genes in response to *P. ultimum*, we analyzed the changes of transcription levels of VPE family genes in response to *P. ultimum* in apple via NCBI-GEO data (GSE62103). The expression levels of MDP0000172104, MDP0000288837, MDP0000188488, MDP0000196515, MDP0000243227, and MDP0000241162 were down-regulated, while the expression levels of MDP0000321943, MDP0000292165, MDP0000759605, MDP0000166283, and MDP0000256408 were up-regulated during the infection of *P. ultimum* (Figure 6A).

ASGV, a kind of latent viruses, is one of the most difficult viruses to remove, in spite of the method of tissue culture were used. Through the analysis of NCBI-GEO database (GSE58293), we found that the expression levels of MDP0000256408, MDP0000122571, MDP0000243227 and MDP0000172014 were down-regulated, and the expression levels of MDP0000084203, MDP0000292815, MDP0000759605, MDP0000241162, MDP0000196515, MDP0000166283, and MDP0000256148 were up-regulated (Figure 6B). Above all, the MDP0000243227 and MDP0000172014 had similar expression patterns under biotic stress including *P. ultimum* and ASGV, while MDP0000759605 and MDP0000166283 were up-regulated. This results indicated *MdVPE* family genes have a diverse roles in responses to biotic stress and MDP0000243227, MDP0000172014, MDP0000759605, and MDP0000166283 may be broad-spectrum disease-resistant genes.

### Expression Analysis of the Selected Genes in Response to Abiotic by qRT-PCR

**FIGURE 7:**
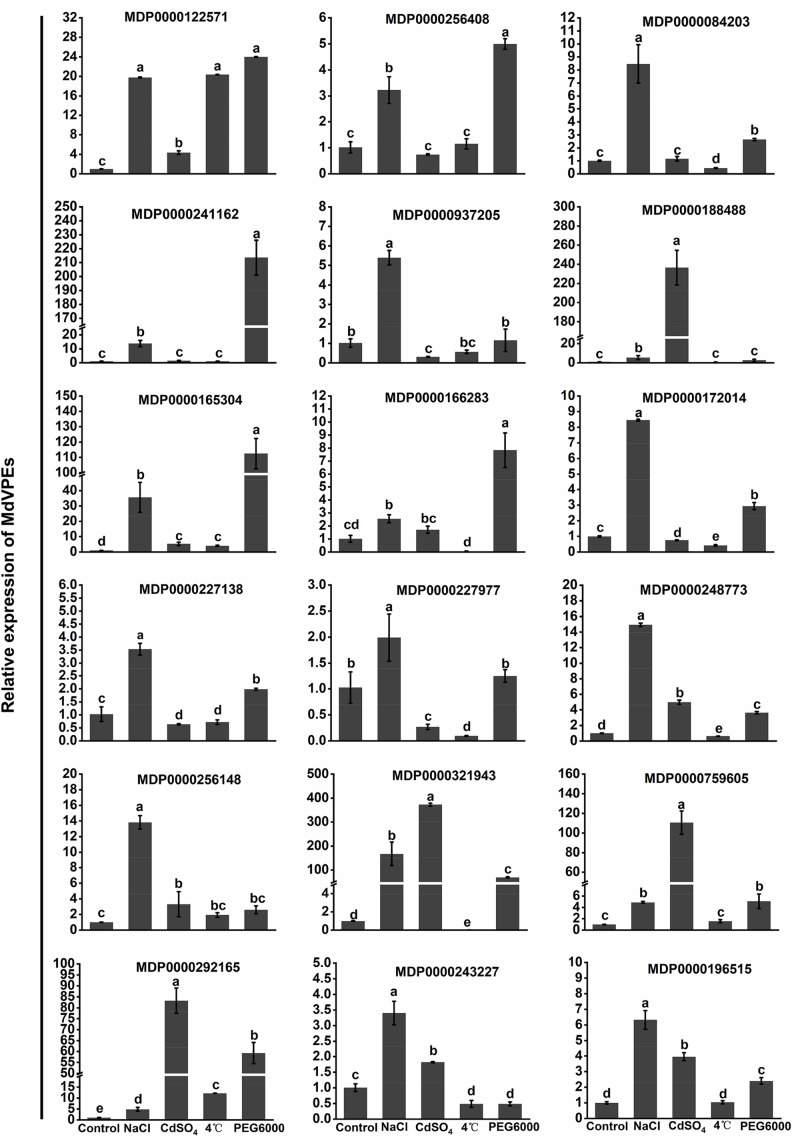
Expression profiles of *MdVPE* genes under salt, cadmium, cold, and drought stresses. Quantitative RT-PCR was used to investigate the expression levels of each *MdVPE* genes in apple roots. The data are presented as means ± standard deviations (SD) of three independent measurements from three individual plants. Means with different small letters in the column are significantly different at *P* < 0.05.

Increasing studies reported that VPEs played an important role in plants response to abiotic stress (Li et al., 2012; Misas-Villamil et al., 2013; Kariya et al., 2018; Locato and De Gara, 2018). Salt, cadmium, low-temperature, and drought stress seriously effected the apple trees growth and development. To reveal the potential function of *MdVPEs* in response to abiotic stress, the expression levels of 18 selected *MdVPE* genes were measured under abiotic stress by qRT-PCR. Considered that the root is the most direct and sensitive organs to feel the changes in the soil environment than the other organs of plants. Vegetative vacuole played a more important role than seed vacuole in the response to stress (Christoff and Rogerio, 2014), and among vegetable organs, fresh roots are rich in vacuoles. Furthermore, most VPEs were predicted to be vacuolar proteins (Table 1). Based on the above views, we selected fresh roots as the samples to investigate the expression patterns of *MdVPE* genes in response to abiotic stress. The expression of all *MdVPEs* were up-regulated under salt stress. Except the down-regulation of MDP0000243227 and the insensitivity of MDP0000188488, MDP0000937205, and MDP0000227977, the other *MdVPEs* were up-regulated under drought stress. Except MDP0000256408, MDP000084203, and MDP0000241162, most of *MdVPEs* were sensitive to cadmium stress. Furthermore, except MDP0000256408, MDP0000241162, MDP0000188488, MDP0000759605, and MDP0000196515, the other *MdVPEs* were sensitive to low-temperature stress. Interestingly, MDP0000084203, MDP0000241162, and MDP0000172014, three *MdVPE* genes belong to *MdγVPEs* (Figure 1), have similar expression patterns, that were more strongly response to drought and salt stress than cadmium and low-temperature stress (Figure 7).

### Overexpression of MDP0000172014 Decreased *Arabidopsis thaliana* Tolerance to Salt Stress

**FIGURE 8:**
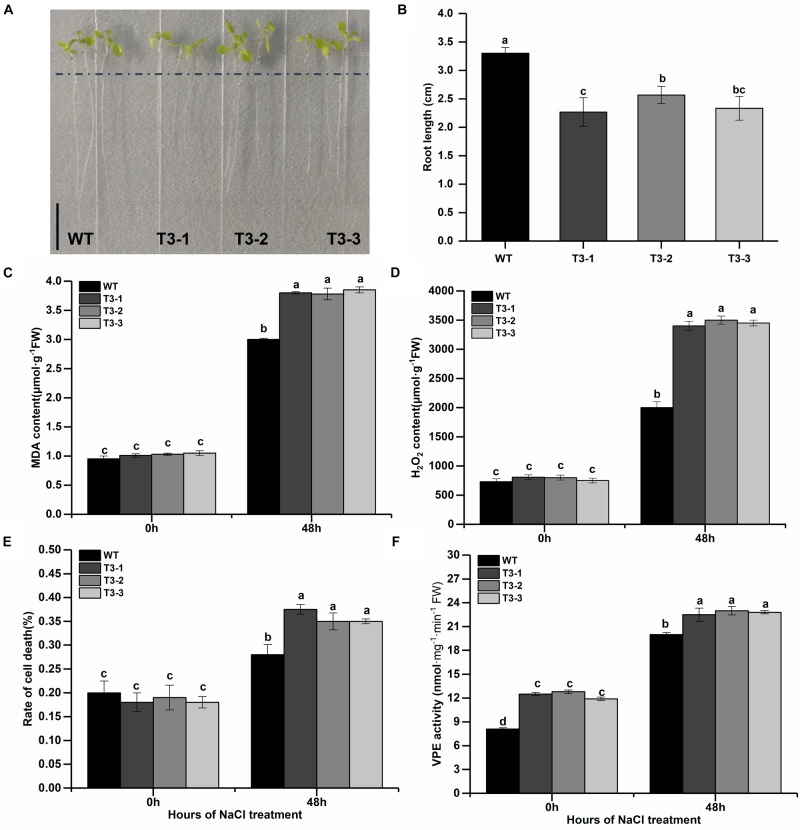
Overexpression of MDP0000172014 decreased the tolerance of *Arabidopsis* to salt stress. **(A)** Phenotype comparisons **(B)** and root length between the wild type (WT) and overexpressed MDP0000172014 transgenic *Arabidopsis* seedlings (T3-1, T3-2, and T3-3) after 100 mM NaCl treatment, and the length of the black line represents 1 cm. **(C)** The MDA, **(D)** H_2_O_2_, **(E)** rate of cell death, and **(F)** VPE activity of WT and transgenic *Arabidopsis* after 200 mM NaCl treatment. The data are presented as means ± standard deviations (SD) of three independent measurements from three individual plants. Means with different small letters in the column are significantly different at *P* < 0.05.

Considered that (1) the salt is the most seriously abiotic stress threated growth and development of apple trees; (2) previous studies had reported that *γVPE* played important role in biotic and abiotic stress resistance (Hatsugai et al., 2004; Christoff et al., 2014; Ran et al., 2014; Su et al., 2015) and the MDP0000172014 belongs to *γVPE* via the analysis of phylogenetic trees (Figure 1); (3) the MDP0000172014 were strongly induced by salt and drought stress, two stress all caused osmotic stress for plants (Figure 7); (4) the *cis*-acting element including w box and ABRE were found in the promoter of MDP0000172014 (Figure 4). To further reveal the MDP0000172014 functions in response to salt stress, we construsted the pBI121-35S-MDP0000172014 vector and transformed it into *Arabidopsis*. The results of Kanamycin screening in T1 and expression analysis of T3 showed that the pBI121-35S-MDP0000172014 vector had been successfully transferred to Col-1 *Arabidopsis thaliana* (Supplementary Figure 2). Moreover, T1 seeds were further screened to T3 on kanamycin resistance medium. Transgenetic lines showed shorter roots length (Figures 8A,B) as well as significantly higher MDA content (Figure 8C), H_2_O_2_ content (Figure 8D) and cell death number (Figure 8E) than control lines. These results suggested that overexpression of MDP0000172014 decreased *Arabidopsis* tolerance to salt stress and promoted salt stress-induced roots cell death.

## Discussion

Increasing studies showed that PCD played a central role in plants developmental process and stress responses (Hatsugai et al., 2015). In animals, the performed of PCD requires caspase cascade including caspase-1 as the initiator and caspase-3 as the executor (Fagundes et al., 2015). Because of VPEs with caspase-1-like activity, it had been known as the key activator of PCD in plants (Hatsugai et al., 2015). Additionally, VPEs composed of a *N*-terminal propeptides (NTPP), a *C*-terminal propeptides (CTPP) and a peptidase_C13 domain (Kuroyanagi et al., 2002), and VPEs also shared high identity in AA sequence (Kuroyanagi et al., 2002). By following criteria, we identified 20 VPEs genes in the apple genome (Table 1). Apple has more members than *Arabidopsis* (four), barley (eight), and tomato (fourteen) (Vorster et al., 2019). Based on ProtComp version 9.0 program, all subcellular localizations of *MdVPE* proteins were predicted, interestingly, MDP0000243227 and MDP0000196515 were located in ER which different from the others, which were located in vacuole. This may suggest that MDP0000243227 and MDP0000196515 had different sites of action from others.

According to homology and functional characteristics, VPEs has been divided into three sub-families, including vegetative VPEs (*γVPE* and *δVPE*), seed VPEs (*αVPE* and β*VPE*) and uncharacterized VPEs (Christoff et al., 2014). The seed VPEs mainly play an important role in plant development and growth, while vegetative VPEs mainly performed functions in stress responses. The NJ phylogenetic trees were constructed for *MdVPEs* with *Arabidopsis*, rice, soybean, and tobacco VPEs, which demonstrated their evolutionary relationships and potential similarities of function (Figure 1, Supplementary Figure 1). Following it, we divided the *MdVPEs* into four groups, including *MdαVPEs*, *MdβVPEs*, *MdγVPEs*, and *MdδVPEs*, and these phylogenetic trees would be the basic information for future study of *MdVPEs*. Considered that genes function were related to their protein structure, the intron-exon structure, motif, and protein structure of *MdVPEs* were analyzed, and these are highly conserved in *MdVPEs*. For example, the homology of MDP0000321943, MDP0000166283, and MDP0000937205 reached over 90%. Moreover, almost all *MdVPEs* have the same motifs in *N* terminal and *C* terminal, which belong to CASc superfamily via the analysis of NCBI-CDD and SMART (Figures 2A–C). However, different and similar motif numbers present in the *MdVPEs* may indicate diverse and similarities functions between various *MdVPEs*.

Increasing studies reported that VPEs involved in plants growth and development, such as participation in tomato sucrose accumulation (Wang et al., 2016), the radish bud abortive (Zhang et al., 2013), and the lack of advantage at the top of the potato tuber (Teper-Bamnolker et al., 2012, 2017). GEO-database (GSE42873) analysis showed the different expression patterns of *MdVPEs* in different tissues, and some of them had similar expression patterns. This may suggest that *MdVPEs* played different roles in different tissues of apple trees and some of them had similar functions. Additionally, some *MdVPEs* had relatively higher expression level in specific tissues (Figure 5), this may indicate them contributed to exercise of function in specific tissues. The important functions of VPE in response to biotic and abiotic stress have been widely reported in pervious study. For example, VPE increased tobacco tolerance to TMV via promoting hypersensitive death (HR) (Hatsugai et al., 2004), and promoted the cell death in plants under heavy mental stress (Kariya et al., 2013, 2018; Ran et al., 2014; Cai et al., 2018). MPK6 promoted PCD of *Arabidopsis* with heat stock treatment though VPEs (Li et al., 2012), Moreover, *GmNAC30* and *GmNAC81* integrate the ER stress-induced cell death responses through *γVPE* (Mendes et al., 2013). Here, GEO database were used to analyze the expression patterns of *MdVPEs* during the infection of *P. ultimum* and ASGV, two diseases hardly removed from apple trees, we found that except MDP0000084203, MDP0000165304, and MDP0000256148, the other *MdVPEs* were involved in *P. ultimum* and ASGV stress response. Among them, *MdγVPEs*, including MDP0000172014 and MDP0000243227 were down-regulated, while MDP0000759605 and MDP0000166283 were up-regulated under the infection of *P. ultimum* and ASGV (Figure 6), and this suggested them may be broad-spectrum disease-resistant genes. Analysis of *cis*-element showed in the promoter of *MdVPEs* had w box, ABRE, LTR, and TC-rich repeats (Figure 4), which indicated *MdVPEs* may response to drought, ABA and low-temperature. Additionally, expression analysis of 18 selected *MdVPEs* showed except MDP0000243227, MDP0000188488, MDP0000937205, and MDP0000227977, the other *MdVPEs* were up-regulated under salt and drought stress. Most *MdVPEs* were sensitive to cadmium and low-temperature stress (Figure 7), which indicated *MdVPEs* may perform important roles in abiotic stress response including salt, cadmium, low-temperature, and drought stress. Mendes et al. (2013) had reported *GmNAC30* and *GmNAC81* mediated osmotic stress-induced cell death through *γVPE*. Interestingly, MDP0000084203, MDP0000241162, and MDP0000172014, three *MdVPE* genes belong to *MdγVPEs* (Figure 1), have similar expression patterns, that were more strongly response to drought and salt stress (all construsted osmotic stress) for plants than cadmium and low-temperature stress (Figure 7). This indicated *MdγVPEs* may play a more central role than other kinds of *MdVPEs* in deafsing to drought and salt stress in apple.

Salt stress seriously threaten the quality and yield of apple. *MdγVPEs* were found may play a more important function under salt stress, and the ABRE was also found in the promoter of MDP0000172014, a *MdγVPE* gene. Therefore, we selected MDP0000172014 as the representative, which was used to reveal the function of MDP0000172014 in response to salt stress. The transgenic *Arabidopsis* of MDP0000172014 were significantly sensitive to salt than wildtype *Arabidopsis* (Figure 8), suggesting that *MdγVPEs* enhanced the sensibility of apple trees to salt stress.

In conclusion, our comprehensive analysis including the identified of *MdVPEs’* characteristics and structure, and the expression analysis under stress, filling the blank of woody plants VPE information and laid a foundation for the further exploration of VPE functions in apple stress resistance. However, the resistance molecular mechanism of them also needed to be further explored.

## Data Availability Statement

The datasets presented in this study can be found in NCBI (https://www.ncbi.nlm.nih.gov/geo/) under accession numbers GSE42873, GSE62103, and GSE53825.

## Author Contributions

JS and WZ designed the experiment. FY and MX performed the MDA and H_2_O_2_ content experiments. LX and XT performed the data analysis. JS drafted the manuscript. WZ revised the manuscript. HY was the project leader. All authors read and approved its final version.

## Conflict of Interest

The authors declare that the research was conducted in the absence of any commercial or financial relationships that could be construed as a potential conflict of interest.

## Footnotes

1 http://pfam.xfam.org/

2 https://phytozome.jgi.doe.gov/pz/portal.html

3 https://www.arabidopsis.org/

4 http://smart.embl-heidelberg.de/

5 https://www.ncbi.nlm.nih.gov/Structure/cdd/wrpsb.cgi

6 http://web.expasy.org/protparam/

7 http://gsds.cbi.pku.edu.cn/index.php

8 http://alternate.meme-suite.org/tools/meme

9 http://linux.softberry.com/

10 http://bioinformatics.psb.ugent.be/webtools/plantcare/html

11 https://www.ncbi.nlm.nih.gov/geo/

## References

[B1] Baskett A. J. (2012). *A Type II Metacaspase Interacts With RPS1-K-2 in Soybean and Analysis of the Soybean Metacaspase Gene Family.* Dissertations Gradworks, Londonderry.

[B2] Cai Y. M. Yu J. Ge Y. Mironov A. Gallois P. (2018). Two proteases with caspase-3-like activity, cathepsin B and proteasome, antagonistically control ER-stress-induced programmed cell death in *Arabidopsis*. *New Phytol.* 218 1143–1155. 10.1111/nph.14676 28675441 PMC5947621

[B3] Cannon S. B. Mitra A. Baumgarten A. Young N. D. May G. (2004). The roles of segmental and tandem gene duplication in the evolution of large gene families in *Arabidopsis thaliana*. *BMC Plant Biol.* 4:10 10.1186/1471-2229-4-10 PMC446195 15171794

[B4] Chen C. J. Xia R. Chen H. He Y. H. (2018). TBtools, a toolkit for biologists integrating various HTS-data2 handling tools with a user-friendly interface. *BioRxiv* [Preprint], 10.1101/069187

[B5] Christoff A. P. Rogerio M. (2014). The diversity of rice phytocystatins. *Mol. Gen. Genomics* 289 1321–1330. 10.1007/s00438-014-0892-7 25098420

[B6] Christoff A. P. Turchetto-Zolet A. C. Margis R. (2014). Uncovering legumain genes in rice. *Plant Sci.* 215-216 100–109. 24388520 10.1016/j.plantsci.2013.11.005

[B7] Cilliers M. Wyk S. G. V. Kunert K. J. Vorster B. J. (2017). Identification and changes of the drought-induced cysteine protease transcriptome in soybean (*Glycine max*) root nodules. *Environ. Exp. Bot.* 148:S0098847217303118.

[B8] Clough S. J. Bent A. F. (1998). Floral dip: a simplified method for Agrobacterium-mediated transformation of *Arabidopsis thaliana*. *Plant J.* 16 735–743. 10.1046/j.1365-313x.1998.00343.x 10069079

[B9] De Pinto M. C. Locato V. De Gara L. (2012). Redox regulation in plant programmed cell death. *Plant Cell Environ.* 35 234–244. 10.1111/j.1365-3040.2011.02387.x 21711357

[B10] Del Pozo O. Lam E. (1998). Caspases and programmed cell death in the hypersensitive response of plants to pathogens. *Curr. Biol.* 8:R896 10.1016/s0960-9822(07)00555-6 9843696

[B11] Deng M. J. Bian H. W. Xie Y. K. Kim Y. H. Wang W. Z. Lin E. (2011). Bcl-2 suppresses hydrogen peroxide-induced programmed cell death via OsVPE2 and OsVPE3, but not via OsVPE1 and OsVPE4, in rice. *FEBS J.* 278 4797–4810. 10.1111/j.1742-4658.2011.08380.x 21972902

[B12] Fagundes D. Bohn B. Cabreira C. Leipelt F. Dias N. Bodanese-Zanettini M. H. (2015). Caspases in plants: metacaspase gene family in plant stress responses. *Funct. Integr. Genomics* 15 639–649. 10.1007/s10142-015-0459-7 26277721

[B13] Gong P. J. Li Y. Tang Y. J. Wei R. Zhu H. Z. Wang Y. J. (2018). Vacuolar processing enzyme (VvbetaVPE) from *Vitis vinifera*, processes seed proteins during ovule development, and accelerates seed germination in VvbetaVPE heterologously over-expressed *Arabidopsis*. *Plant Sci.* 274 420–431. 10.1016/j.plantsci.2018.06.023 30080630

[B14] Hatsugai N. Kuroyanagi M. Yamada K. Meshi T. Tsuda S. Kondo M. (2004). A plant vacuolar protease, VPE, mediates virus-induced hypersensitive cell death. *Science* 305 855–858. 10.1126/science.1099859 15297671

[B15] Hatsugai N. Yamada K. Goto-Yamada S. Hara-Nishimura I. (2015). Vacuolar processing enzyme in plant programmed cell death. *Front. Plant Sci.* 6:234 10.3389/fpls.2015.00234 PMC4390986 25914711

[B16] Hu B. Jin J. Guo A. Y. Zhang H. Luo J. Gao G. (2015). GSDS 2.0*: an upgraded gene feature visualization server*. *Bioinformatics* 31 1296–1297. 10.1093/bioinformatics/btu817 25504850 PMC4393523

[B17] Jiang J. J. Hu J. Z. Tan R. J. Han Y. H. Li Z. Y. (2019). Expression of IbVPE1 from sweet potato in *Arabidopsis* affects leaf development, flowering time and chlorophyll catabolism. *BMC Plant Biol.* 19:184 10.1186/s12870-019-1789-8 PMC6503384 31060496

[B18] Kariya K. Demiral T. Sasaki T. Tsuchiya Y. Turkan I. Sano T. (2013). A novel mechanism of aluminum-induced cell death involving vacuolar processing enzyme and vacuolar collapse in tobacco cell line BY-2. *J. Inorg. Biochem.* 128 196–201. 10.1016/j.jinorgbio.2013.07.001 23891542

[B19] Kariya K. Tsuchiya Y. Sasaki T. Yamamoto Y. (2018). Aluminium-induced cell death requires upregulation of NtVPE1 gene coding vacuolar processing enzyme in tobacco (*Nicotiana tabacum* L.). *J. Inorg. Biochem.* 181 152–161. 10.1016/j.jinorgbio.2017.09.008 28967473

[B20] Kim Y. Wang M. Q. Bai Y. Zeng Z. H. Guo F. Han N. (2014). Bcl-2 suppresses activation of VPEs by inhibiting cytosolic Ca(2)(+) level with elevated K(+) efflux in NaCl-induced PCD in rice. *Plant Physiol. Biochem.* 80 168–175. 10.1016/j.plaphy.2014.04.002 24787501

[B21] Kumar S. Stecher G. Tamura K. (2016). MEGA7: molecular evolutionary genetics analysis version 7.0 for bigger datasets. *Mol. Biol. Evol.* 33 1870–1874. 10.1093/molbev/msw054 27004904 PMC8210823

[B22] Kuroyanagi M. Nishimura M. Hara-Nishimura I. (2002). Activation of *Arabidopsis* vacuolar processing enzyme by self-catalytic removal of an auto-inhibitory domain of the C-terminal propeptide. *Plant Cell Physiol.* 43 143–151. 10.1093/pcp/pcf035 11867693

[B23] Kuroyanagi M. Yamada K. Hatsugai N. Kondo M. Nishimura M. Hara-Nishimura I. (2005). Vacuolar processing enzyme is essential for mycotoxin-induced cell death in *Arabidopsis thaliana*. *J. Biol. Chem.* 280 32914–32920. 10.1074/jbc.M504476200 16043487

[B24] Lam E. Zhang Y. (2012). Regulating the reapers: activating metacaspases for programmed cell death. *Trends Plant Sci.* 17 487–494. 10.1016/j.tplants.2012.05.003 22658651

[B25] Lamkanfi M. Declercq W. Kalai M. Saelens X. Vandenabeele P. (2002). Alice in caspase land. A phylogenetic analysis of caspases from worm to man. *Cell Death Differ.* 9 358–361. 10.1038/sj.cdd.4400989 11965488

[B26] Landi M. (2017). Commentary to: Improving the thiobarbituric acid-reactive-substances assay for estimating lipid peroxidation in plant tissues containing anthocyanin and other interfering compounds by Hodges et al., Planta (1999) 207:604-611. *Planta* 245:1067 10.1007/s00425-017-2699-3 28456836

[B27] Li Z. Yue H. Y. Xing D. (2012). MAP Kinase 6-mediated activation of vacuolar processing enzyme modulates heat shock-induced programmed cell death in *Arabidopsis*. *New Phytol.* 195 85–96. 10.1111/j.1469-8137.2012.04131.x 22497243

[B28] Livak K. J. Schmittgen T. D. (2001). Analysis of relative gene expression data using real-time quantitative PCR and the 2(-Delta Delta C(T)) Method. *Methods* 25 402–408. 10.1006/meth.2001.1262 11846609

[B29] Locato V. De Gara L. (2018). Programmed cell death in plants: an overview. *Methods Mol. Biol.* 1743 1–8. 10.1007/978-1-4939-7668-3_1 29332281

[B30] Mao K. Dong Q. L. Li C. Liu C. H. Ma F. W. (2017). Genome wide identification and characterization of apple bHLH transcription factors and expression analysis in response to drought and salt stress. *Front. Plant. Sci.* 8:480 10.3389/fpls.2015.00480 PMC5387082 28443104

[B31] Marchler-Bauer A. Bo Y. Han L. He J. Lanczycki C. J. Lu S. (2017). CDD/SPARCLE: functional classification of proteins via subfamily domain architectures. *Nucleic Acids Res.* 45 D200–D203. 10.1093/nar/gkw1129 27899674 PMC5210587

[B32] Mendes G. C. Reis P. A. Calil I. P. Carvalho H. H. Aragao F. J. Fontes E. P. (2013). GmNAC30 and GmNAC81 integrate the endoplasmic reticulum stress- and osmotic stress-induced cell death responses through a vacuolar processing enzyme. *Proc. Natl. Acad. Sci. U.S.A.* 110 19627–19632. 10.1073/pnas.1311729110 24145438 PMC3845183

[B33] Meng D. Li Y. Y. Bai Y. Li M. J. Cheng L. L. (2016). Genome-wide identification and characterization of WRKY transcriptional factor family in apple and analysis of their responses to waterlogging and drought stress. *Plant Physiol. Biochem.* 103 71–83. 10.1016/j.plaphy.2016.02.006 26970718

[B34] Misas-Villamil J. C. Toenges G. Kolodziejek I. Sadaghiani A. M. Kaschani F. Colby T. (2013). Activity profiling of vacuolar processing enzymes reveals a role for VPE during oomycete infection. *Plant J.* 73 689–700. 10.1111/tpj.12062 23134548

[B35] Patterson B. D. Macrae E. A. Ferguson I. B. (1984). Estimation of hydrogen peroxide in plant extracts using titanium(IV). *Anal. Biochem.* 139 487–492. 10.1016/0003-2697(84)90039-3 6476384

[B36] Radchuk V. Tran V. Radchuk R. Diaz-Mendoza M. Weier D. Fuchs J. (2018). Vacuolar processing enzyme 4 contributes to maternal control of grain size in barley by executing programmed cell death in the pericarp. *New Phytol.* 218 1127–1142. 10.1111/nph.14729 28836669

[B37] Ran K. Yang H. Q. Sun X. L. Li Q. Jiang Q. Q. Zhang W. W. (2014). Isolation, characterization, and structure analysis of a vacuolar processing enzyme gene (MhVPEgamma) from *Malus hupehensis* (Pamp) Rehd. *Appl. Biochem. Biotechnol.* 173 579–595. 10.1007/s12010-014-0867-5 24691880

[B38] Rojo E. Martin R. Carter C. Zouhar J. Pan S. Plotnikova J. (2004). VPEgamma exhibits a caspase-like activity that contributes to defense against pathogens. *Curr. Biol.* 14 1897–1906. 10.1016/j.cub.2004.09.056 15530390

[B39] Shimada T. Takagi J. Ichino T. Shirakawa M. Hara-Nishimura I. (2018). Plant Vacuoles. *Annu. Rev. Plant Biol.* 69 123–145. 29561663 10.1146/annurev-arplant-042817-040508

[B40] Shimada T. Yamada K. Kataoka M. Nakaune S. Koumoto Y. Kuroyanagi M. (2003). Vacuolar processing enzymes are essential for proper processing of seed storage proteins in *Arabidopsis thaliana*. *J. Biol. Chem.* 278 32292–32299. 10.1074/jbc.M305740200 12799370

[B41] Su Q. Ran K. Men X. J. Zhang W. W. Fan S. L. Yan L. J. (2015). Response of vacuolar processing enzyme in Malus hupehensis and MhVPEγ -overexpressing *Arabidopsis* to high temperature stress. *Acta Physiol. Plant.* 37:82.

[B42] Teper-Bamnolker P. Buskila Y. Belausov E. Wolf D. Doron-Faigenboim A. Ben-Dor S. (2017). Vacuolar processing enzyme activates programmed cell death in the apical meristem inducing loss of apical dominance. *Cell* 40 2381–2392. 10.1111/pce.13044 28755442

[B43] Teper-Bamnolker P. Buskila Y. Lopesco Y. Ben-Dor S. Saad I. Holdengreber V. (2012). Release of apical dominance in potato tuber is accompanied by programmed cell death in the apical bud meristem. *Plant Physiol.* 158 2053–2067. 10.1104/pp.112.194076 22362870 PMC3320206

[B44] Thompson J. D. Gibson T. J. Plewniak F. Jeanmougin F. Higgins D. G. (1997). The CLUSTAL_X windows interface: flexible strategies for multiple sequence alignment aided by quality analysis tools. *Nucleic Acids Res.* 25 4876–4882. 10.1093/nar/25.24.4876 9396791 PMC147148

[B45] Tsiatsiani L. Van Breusegem F. Gallois P. Zavialov A. Lam E. Bozhkov P. V. (2011). Metacaspases. *Cell Death. Differ.* 18 1279–1288. 21597462 10.1038/cdd.2011.66 PMC3172103

[B46] Van Wyk S. G. Du Plessis M. Cullis C. A. Kunert K. J. Vorster B. J. (2014). Cysteine protease and cystatin expression and activity during soybean nodule development and senescence. *BMC Plant Biol.* 14:294 10.1186/s12870-014-0294-3 PMC4243279 25404209

[B47] Vartapetian A. B. Tuzhikov A. I. Chichkova N. V. Taliansky M. Wolpert T. J. (2011). A plant alternative to animal caspases: subtilisin-like proteases. *Cell Death. Differ.* 18 1289–1297. 10.1038/cdd.2011.49 21546909 PMC3172098

[B48] Velasco R. Zharkikh A. Affourtit J. Dhingra A. Cestaro A. Kalyanaraman A. (2010). The genome of the domesticated apple (Malus x domestica Borkh.). *Nat. Genet.* 42 833–839. 10.1186/s12864-015-2096-x 20802477

[B49] Vorster B. J. Cullis C. A. Kunert K. J. (2019). Plant vacuolar processing enzymes. *Front. Plant Sci.* 10:479 10.3389/fpls.2015.00479 PMC6473326 31031794

[B50] Wang N. Duhita N. Ariizumi T. Ezura H. (2016). Involvement of vacuolar processing enzyme SlVPE5 in post-transcriptional process of invertase in sucrose accumulation in tomato. *Plant Physiol. Biochem.* 108 71–78. 10.1016/j.plaphy.2016.06.037 27423072

[B51] Wang W. Xiong H. Y. Lin R. X. Zhao N. T. Zhao P. Sun M. X. (2019). A VPE-like protease NtTPE8 exclusively expresses in the integumentary tapetum and is involved in seed development. *J. Integr. Plant Biol.* 61 598–610. 10.1111/jipb.12766 30589207

[B52] Wang Y. H. Zhu S. S. Liu S. J. Jiang L. Chen L. M. Ren Y. L. (2009). The vacuolar processing enzyme OsVPE1 is required for efficient glutelin processing in rice. *Plant J.* 58 606–617. 10.1111/j.1365-313X.2009.03801.x 19154227

[B53] Woltering E. J. Van Der Bent A. Hoeberichts F. A. (2002). Do plant caspases exist? *Plant Physiol.* 130 1764–1769. 10.1104/pp.006338 12481059 PMC1540272

[B54] Yamada K. Basak A. K. Goto-Yamada S. Tarnawska-Glatt K. Hara-Nishimura I. (2019). Vacuolar processing enzymes in the plant life cycle. *New Phytol.* 226 21–31. 10.1111/nph.16306 31679161

[B55] Zhang J. Li Q. F. Huang W. W. Xu X. Y. Zhang X. L. Hui M. X. (2013). A vacuolar processing enzyme RsVPE1 gene of radish is involved in floral bud abortion under heat stress. *Int. J. Mol. Sci.* 14 13346–13359. 10.3390/ijms140713346 23807498 PMC3742190

[B56] Zhang W. W. Song J. F. Yue S. Q. Duan K. X. Yang H. Q. (2019). MhMAPK4 from Malus hupehensis Rehd. decreases cell death in tobacco roots by controlling Cd(2+) uptake. *Ecotoxicol. Environ. Saf.* 168 230–240. 10.1016/j.ecoenv.2018.09.126 30388541

