# Supplementary material for: Genome-Wide Identification and Characterization of Vacuolar Processing Enzyme Gene Family and Diverse Expression Under Stress in Apple (Malus × Domestic)
Source: Front Plant Sci. 2020 May 26;11:626. doi: 10.3389/fpls.2020.00626 (PMC7264823; doi:10.3389/fpls.2020.00626)
Supplement: Supplementary file 1 [file Data_Sheet_1.PDF]

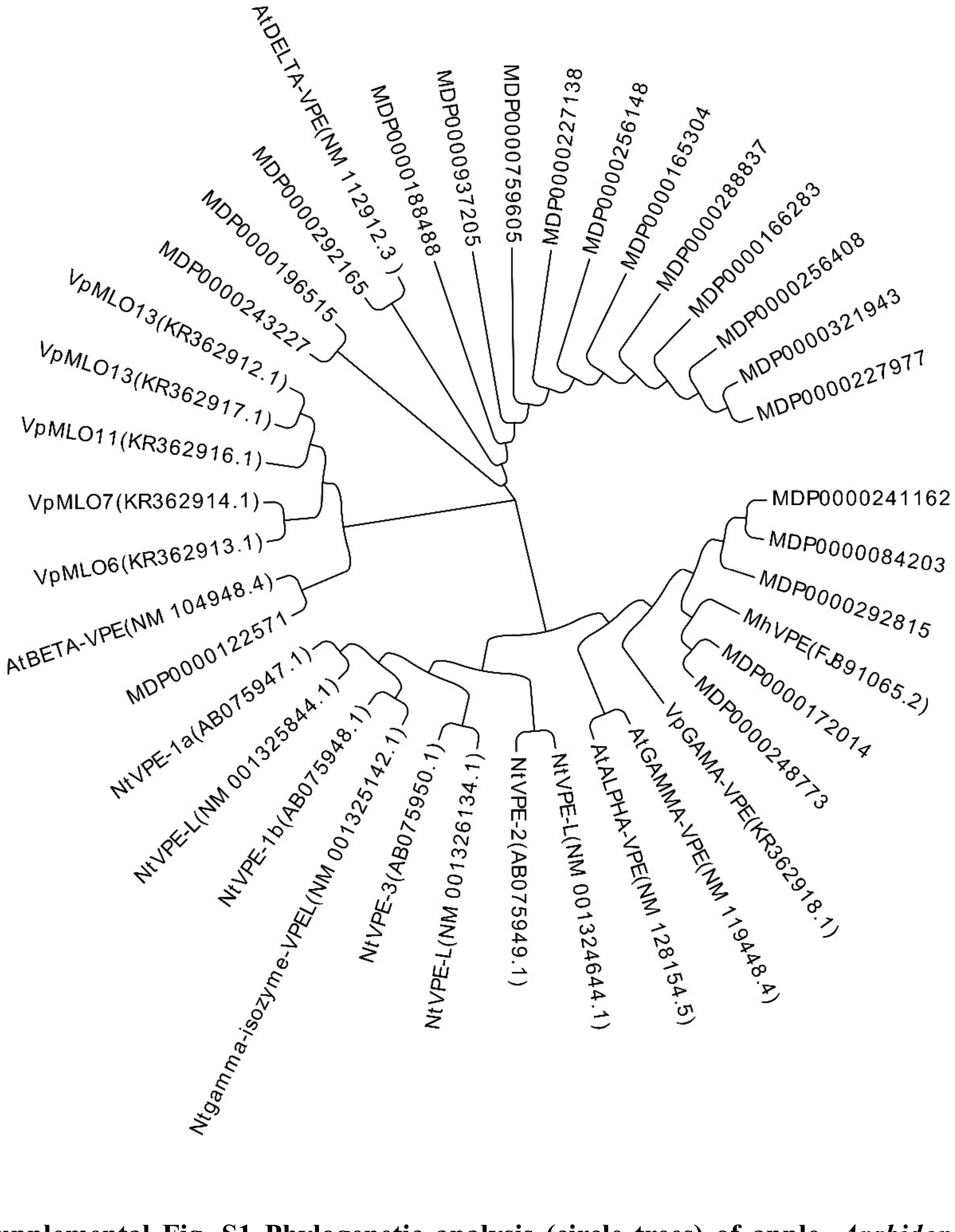

Supplemental Fig. S1 Phylogenetic analysis (circle trees) of apple, *Arabidopsis*, tobocca, soybean, and *Vitis pseudoreticulata* VPE proteins. Phylogenetic trees were contrusted by neighbor-joining method with bootstrap values 1000 via MEGA 7.0.

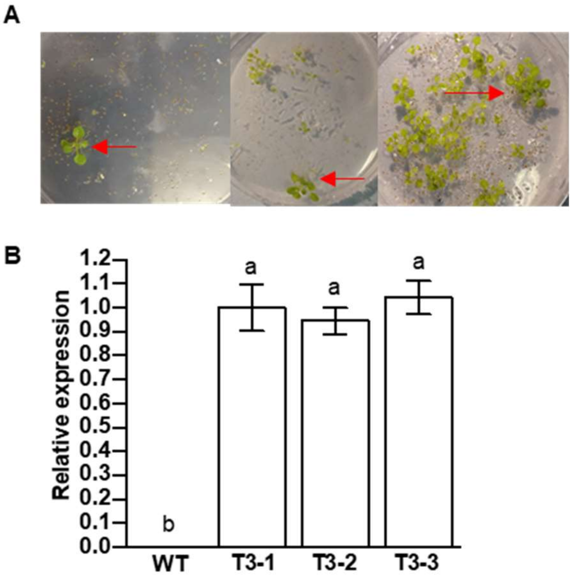

Supplemental Fig. S2 The transformation of MDP0000172014 into Col-1 *Arabidopsis*. (A) Rapid screening of kanamycin resistance of T1 transgenic *Arabidopsis*. As the arrowhead showed, the seedings with green leaves is kanamycin-resistant seedlings. (B) The relative expression of MDP0000172014 in wildtype (WT) and transgenic *Arabidopsis* (T3). Quantitative RT-PCR was used to investigate the expression levels of each *MdVPE* genes in apple wildtype (WT) and transgenic *Arabidopsis* (T3).
