# Supplementary material for: Genome-Wide Identification and Characterization of Vacuolar Processing Enzyme Gene Family and Diverse Expression Under Stress in Apple (Malus × Domestic)
Source: Front Plant Sci. 2020 May 26;11:626. doi: 10.3389/fpls.2020.00626 (PMC7264823; doi:10.3389/fpls.2020.00626)
Supplement: Supplementary file 2 [file Table_1.docx]

**Supplenmental Table 1** The primes for qRT-PCR and vector construction of *MdVPE* genes

| Gene ID | Forward primes | Reverse primes | Usage |
| --- | --- | --- | --- |
| qMDP0000122571 | CGAGGCCGGGAGTTATCATC | CATGGCGACCTGATCCTCTC | qRT-PCR |
| qMDP0000256408 | GGGCAGACCTGAAAGAACCT | TGCACGAATACCTCCCATCG |  |
| qMDP0000084203 | GTCATCATTAACAGCCCGCAT | CTTCCCACTACCCCCTGTAA |  |
| qMDP0000241162 | GGGTAGTGGGAAGGTTGTGG | TGCATAGACTTGATATCTCCAAGAA |  |
| qMDP0000937205 | AAGGTTCTGCTTAGCGGTCC | CGCCATCAGTAGGCATAGCA |  |
| qMDP0000188488 | TAAGCCAGATGGCCCTGATG | CCCCGCTGAGAGCTGTTTTA |  |
| qMDP0000165304 | TGGATGCCCAGCGATTATGT | GCTTCGAACTTGAAAGGAGGC |  |
| qMDP0000166283 | CGCGTACAACTCGGAAAATCC | CCTCCGGTGAGAGCACTTTT |  |
| qMDP0000172014 | CAGCCGCTTGTTGATGACTG | GCCATCTGCTCCTGTGTCAT |  |
| qMDP0000227138 | GGAGGAAGTGGCAAGGTTCT | TCTTCTTCGAACCATGCTTTTGT |  |
| qMDP0000227977 | ATGTCGATTGAATTACAGGTTCG | ATCGCTGGTGGAAGTATGGC |  |
| qMDP0000248773 | AGGCCCACAAGTACTCAACG | GTTGCAGATGTTTGCCAGGG |  |
| qMDP0000256148 | TGAGGAGTGGCGAAATGGAA | ATTGCCGTCACCTTCTCGTT |  |
| qMDP0000321943 | GCTACCACTGCGTCAAATGC | ACTTATGTCACTGCAATCCTCC |  |
| qMDP0000759605 | CAAGGGCTCTTCACGTACCA | TGGCATATGTCAGCCTGGTG |  |
| qMDP0000292165 | GTGAAATGGCACTCTCACGC | TAAATGGTCCCCGGTCCAAC |  |
| qMDP0000243227 | GGCGGTGGCTGCGTATTT | ATCGAGAGGTGCAGACCAGA |  |
| qMDP0000196515 | GCTTGTTGATGTCAACCGCA | TTGGTACCAGTTGCAGGTGT |  |
| Md-actin | TAAGGCTGGATTTGCTGGAG | GCATCTTTCTGACCCATTCC |  |
| At-18S | TTGCGTTTGAGAGGATGTGG | TCCATAAGTCGGGGTTTGTTG |  |
| MDP0000172014 | CACCATCACGCCATGGTCGACATGACTCGGTTGGCCAGCG | TCTATCGATCAATCAGGATCCTCACGCACTGAATCCCCG | vector construction |
